# New developments in the study of the microbiota of raw-milk, long-ripened cheeses by molecular methods: the case of Grana Padano and Parmigiano Reggiano

**DOI:** 10.3389/fmicb.2013.00036

**Published:** 2013-02-28

**Authors:** Erasmo Neviani, Benedetta Bottari, Camilla Lazzi, Monica Gatti

**Affiliations:** Department of Food Science, University of ParmaParma, Italy

**Keywords:** long-ripened cheeses microbiota, raw-milk, whey starter, culture-dependent methods, culture-independent methods, starter lactic acid bacteria, non-starter lactic acid bacteria

## Abstract

Microorganisms are an essential component of cheeses and play important roles during both cheese manufacture and ripening. Both starter and secondary flora modify the physical and chemical properties of cheese, contributing and reacting to changes that occur during the manufacture and ripening of cheese. As the composition of microbial population changes under the influence of continuous shifts in environmental conditions and microorganisms interactions during manufacturing and ripening, the characteristics of a given cheese depend also on microflora dynamics. The microbiota present in cheese is complex and its growth and activity represent the most important, but the least controllable steps. In the past, research in this area was dependent on classical microbiological techniques. However, culture-dependent methods are time-consuming and approaches that include a culturing step can lead to inaccuracies due to species present in low numbers or simply uncultivable. Therefore, they cannot be used as a unique tool to monitor community dynamics. For these reasons approaches to cheese microbiology had to change dramatically. To address this, in recent years the focus on the use of culture-independent methods based on the direct analysis of DNA (or RNA) has rapidly increased. Application of such techniques to the study of cheese microbiology represents a rapid, sound, reliable, and effective way for the detection and identification of the microorganisms present in dairy products, leading to major advances in understanding this complex microbial ecosystem and its impact on cheese ripening and quality. In this article, an overview on the recent advances in the use of molecular methods for thorough analysis of microbial communities in cheeses is given. Furthermore, applications of culture-independent approaches to study the microbiology of two important raw-milk, long-ripened cheeses such as Grana Padano and Parmigiano Reggiano, are presented.

## INTRODUCTION

Grana Padano (GP) and Parmigiano Reggiano (PR), the most widespread Italian cheese varieties, are raw-milk, long-ripened, hard cooked cheeses. These traditional cheeses have always been pre-eminent over all Italian dairy productions, holding a remarkable position with respect to food industry economy, and Italian civilization and history.

The origin of these two artisanal cheeses date back to 1200–1300 a.C., thus their production technology has developed throughout more than 700 years of history. They are both produced in Northern Italy, but in two different specific geographic areas, divided by Po River, defined by protected designation of origin (PDO).

**FIGURE 1:**
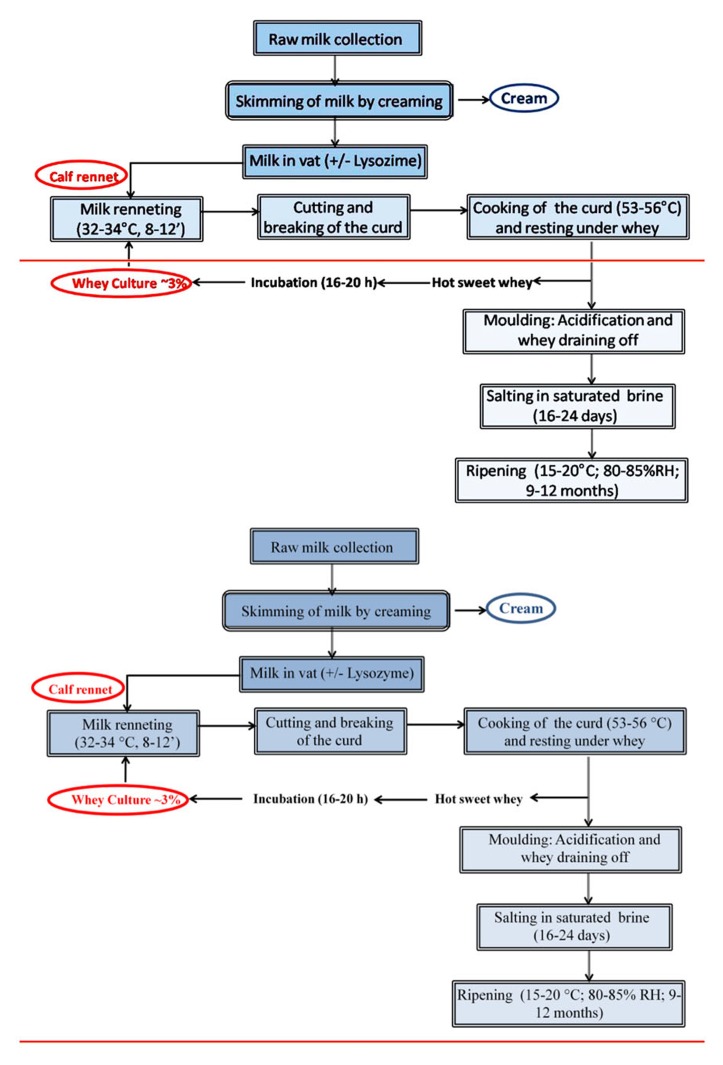
**Grana Padano (GP) and Parmigiano Reggiano (PR) cheese-making technology**.

The transformation of milk into GP and PR cheese concerns a combination of milk, rennet, microorganisms, and salt, which are processed together through different steps, including whey expulsion, acid production, salt addition, and ripening. The layout of productions of the two cheeses^1,2^ are very similar and are synthesized in **Figure 1** (Neviani and Carini, 1994; Mucchetti and Neviani, 2006).

parmigiano Reggiano is produced exclusively in a geographical area hosting farms where the cattle are fed on locally grown forage. The use of silage and fermented feeds is not allowed. For the cheese-making a mixture of milk from two consecutive milking is used. Only non-refrigerated raw milk is used for cheese production. The spontaneous milk creaming of evening milking occurs overnight at room temperature (according to the season) in large tanks called “bacinella,” with traditional capacity of about 1000–1200 l. Cream rising permits to separate partially skimmed milk that will be mixed in a ratio about 1:1 with the whole morning milk. For the cheese-making, traditional overturned shaped bell copper vats with the capacity of about 1200 l are used. The form of these particular vats facilitate heat exchanges. The fat content of vat milk ranges between 2.4 and 2.8%.

Differently from PR in the production of GP the use of high quality silage and fermented feeds is permitted for cow feeding. Use of silage frequently involves the presence of some spore-forming bacteria in raw milk. For this reason and to inhibit late blowing of cheese, caused by butyric fermentations, the addition of lysozyme to the vat milk (20–30 ppm) is allowed. Raw milk refrigeration is permitted and GP must be made from raw cow’s milk which is stored at 8°C at the farm. Milk of each single milking is used for cheese-making. Also in this case milk is naturally creamed in “bacinella.”

Both for PR and GP the use of a natural whey starter, added to the vat milk, permits to increase lactic acid bacteria (LAB) population and acidity of the vat milk. Milk coagulation occurs by adding calf rennet powder (highly purified chymosin preparation) to raw milk which is heated at 32–34°C. The curd reaches the proper firmness and then is broken down into little granules, of about 2–4 mm, and heated up to 53–56°C under stirring. Cooking coupled with the acidity, closed to 4.0–4.3°SH/50, reached adding the natural whey starter, promotes the formation of the proper texture of curd granules and whey drainage. At the end of heating, stirring is turned off and the curd granules aggregate together at the bottom of the vat. The name of “grana,” traditionally given to these two cheeses by Italian people, was due to the particular structure of cheese paste and, particularly, to the characteristic grainy texture obtained. In this technological phase curd rest under the whey at a temperature closed to the one reached at the end of the cooking process.

Curd is removed from vat and cut in two portions that are placed in a mold. After a 2-day molding, having now formed a cylindrical shape, cheese is salted by immersion in saturated salt brine for about 3 weeks, depending both on the concentration of saline solution and on the size of the wheels. Then, the salted cheese is kept in the ripening room. Throughout ripening, that lasts at minimum 9 and 12 months, respectively for GP and PR, cheese undergoes a series of physical, chemical, and microbiological changes which are reflected in its organoleptic characteristics. At the end of ripening, from each vat, two cheeses, each weighing around 35 kg, are obtained. They are hard granular cheeses highly concentrated that contain only about 30% water and 70% nutrients.

Grana Padano and Parmigiano Reggiano have a specific microbial setup, which plays a crucial technological role. GP and PR manufacturing involves dynamic microbial communities of LAB. LAB, from raw milk and natural whey starter (undefined thermophilic starter culture in whey), have a crucial role during both cheese-making and ripening. Several biochemical reactions occur, largely based on glycolytic, and proteolytic activities. The most complex and most important biochemical event taking place during maturation is undoubtedly proteolysis. It contributes to cheese texture formation by breaking down milk proteins, it decreases the water activity through water binding by increasing the free carboxylic and amino groups, and it promotes flavor development both directly [through the release of mostly bitter peptides and amino acids (AAs)] and indirectly by producing substrates for other biochemical reactions (such as AAs deamination and decarboxylation).

Survival, growth, and biochemical activity of microorganisms in cheese are connected to the bacteria stress response to physical and chemical changes in the curd-cheese environment. We can say that LAB, from raw milk and natural starter, are selected from the same cheese environment, that they themselves contribute to change during ripening.

## MOLECULAR APPROACHES FOR ANALYZING RAW-MILK, LONG-RIPENED CHEESES MICROBIAL COMPOSITION

Different techniques are available today to catch the entire diversity of microbial community. In general, two kinds of approach could be reported: culture-dependent and culture-independent. The culture-dependent approach relies on growing bacteria and implies the following steps including: (i) growing of bacteria on microbiological media before enumeration, (ii) isolation, (iii) identification at genus and species level, and (iv) characterization of biotypes at intraspecific level.

In the case of dairy products, the de Man, Rogosa, Sharpe (MRS; de Man et al., 1960) medium is the most suitable for the isolation of LAB, however, there have been some attempts to devise suites of culture media to maximize the recovery of diverse microbial groups from GP and PR cheese (Gatti et al., 2003; Fornasari et al., 2006; Neviani et al., 2009). Traditional cultural media aimed to recover the majority of microorganisms could be too generic and not selective enough to differentiate species which are present in different amounts. Nutritionally complete media may not allow the count of all microbial populations present in minority in raw-milk, long-ripened cheeses such as GP and PR: i.e., some LAB species such as *Staphylococcus *(*S.*)* thermophilus*. For this reason, Fornasari et al. (2006) proposed to restore the cultivability of *S. thermophilus* by a short pre-incubation of the starters in sterile skimmed whey, followed by cultivation in sterile skimmed whey-enriched M17. This procedure is a useful strategy to isolate *S. thermophilus* from GP natural whey cultures. The employment of not acidified fresh whey in cultured medium (whey agar medium, WAM) has been reported by Gatti et al. (2003) for counts and isolation of streptococci and thermophilic lactobacilli typical of natural whey starters for PR cheese.

To recover the minority microbiota, in the early stages of cheese production (less than 10^2^ cfu/g), Neviani et al. (2009) reported the use of a cheese-based medium (cheese agar medium, CAM). This medium, formulated with 24 months grated PR cheese, promotes the microbiota that better adapts to the changes in nutritional availability and technological parameters occurring during production and ripening. In particular CAM, apart from being an optimal medium to follow the microbial succession in PR during ripening, it’s also able to recover *Lactobacillus *(*Lb.*)* rhamnosus* species. This species, that dominate in the cheese from the 2nd to the 20th month, is hardly estimable on traditional media in the earlier stages when it is minority. Regardless of the culture medium used, the next steps after bacterial count concern the isolation, that is carried out by a random selection of colonies resulting different in the morphology and color. Afterward, by different techniques, the microbial isolates are identified at species level and typed at intraspecific level. The approaches to analyze the microbial diversity can involve both phenotyping and genotyping methods. Traditionally LAB have been classified on the basis of their phenotypical properties but these methods have drawbacks that adversely affect the identification at the genus or species level, such as poor reproducibility and poor accuracy. Moreover physiological and biochemical tests are often not adequately efficient to discriminate at strain level because the bacterial population often has similar nutritional requirements and grows under similar environmental conditions. Thus the main focus for identification has moved from phenotypical to genotypical methods as the latter generate more sensitive and accurate results.

Lactic acid bacteria species are genetically identified by species-specific polymerase chain reaction (PCR; Tilsala-Timisjärvi and Alatossava, 1997; Chagnaud et al., 2001), amplified ribosomal DNA restriction analysis (16S-ARDRA; Roy et al., 2001; Bouton et al., 2002), and sequencing of 16S ribosomal RNA (rRNA) gene (Vásquez et al., 2005). A specific ARDRA [tRNAAla-23S rDNA-restriction fragment length polymorphism (RFLP)] was recently applied by Mancini et al. (2012), to identify isolates collected during the production and ripening of GP cheese. This method, based on a restriction of a specific fragment of 16S–23S rDNA, represents a valid option for dairy LAB screening and identification.

Furthermore, more recently, other target genes than 16S rRNA have been proposed for a reliable distinction of closely related species present in a specific dairy ecosystem such as acidified natural whey (Cremonesi et al., 2011). In a recent work by Bottari et al. (2013), the gene phenylalanine synthase (*pheS*), encoding phenylalanyl-tRNA synthase, was used as a molecular target to design species-specific primers for multiplex real-time PCR (mRealT-PCR) to detect thermophilic LAB in 29 natural whey starters for PR cheese production.

The method proposed by the authors is rapid and effective for analyzing species present in the samples with an average sensitivity down to less than 600 copies of DNA and therefore sensitive enough to detect even minor LAB community members of thermophilic starter cultures.

Given the importance of LAB as the predominant microbiota during cheese-making, the last decade has seen an increasing number of studies not only aimed at identifying them at the species level but also aimed to characterize the specific biotypes at subspecies level.

So, several molecular typing techniques have been applied extensively for genotyping of LAB isolated during manufacturing and ripening of cheese. The most powerful of these are DNA fingerprinting techniques: RFLP of protein-coding genes involved in primary metabolism (Giraffa et al., 2003) and 16S rRNA (Gala et al., 2008), restriction enzyme analysis pulsed-field gel electrophoresis (REA-PFGE; Giraffa et al., 2004; Herrero-Fresno et al., 2012), randomly amplified polymorphic DNA (RAPD; Ercolini et al., 2009; Franciosi et al., 2009; Bove et al., 2011), amplified fragment length polymorphism (AFLP; Lazzi et al., 2009), and repetitive extragenic palindromic-PCR (REP-PCR; Berthier et al., 2001; Bove et al., 2011; Mancini et al., 2012; Solieri et al., 2012). A different discriminatory power was found between these techniques. Among all, RAPD is considered a fast method widely used for the characterization of bacteria, and specifically for the characterization of LAB in dairy products (De Angelis et al., 2001; Andrighetto et al., 2004; Giraffa and Rossetti, 2004; Aquilanti et al., 2007; Rossetti et al., 2008; Randazzo et al., 2009; Bove et al., 2011; Monfredini et al., 2012). Nevertheless this technique has two significant drawbacks: (i) a low discriminatory power (ii) a low reproducibility, both between and within laboratories, as reported by different authors (Power, 1996; Tyler et al., 1997). In comparison, REP-PCR is more reproducible because specific primers are used for amplification; however, also REP-PCR has disadvantages including potential contamination, artifacts, and the need for multiple controls (Li et al., 2009). Studies comparing genotyping methods highlighted that AFLP showed a higher discriminatory power than RAPD (Clerc et al., 1998). Recently, Di Cagno et al. (2010) demonstrated that the values of the D index for RAPD-PCR and AFLP analyses were 0.92 and 0.99, respectively. This confirms the view taken by Lazzi et al. (2009): AFLP provides a better view of genotypic heterogeneity within the strains of the same species.

In the case of PR and GP, several investigations were carried out to improve the knowledge of the taxonomic diversity and heterogeneity mainly of natural whey starter microbiota.

Several researches were carried out to determine the microbial species that characterize this ecosystem (Rossetti et al., 2008; Bottari et al., 2013), their physiological role and their resistance to different antibiotics (Belletti et al., 2009; Rossetti et al., 2009). In particular, studies have focused on the biodiversity of *Lb. helveticus*, the dominant species among the thermophilic microbiota of natural whey cultures (Gatti et al., 2003, 2004a; Andrighetto et al., 2004; Rossetti et al., 2008).

Since *Lb. helveticus* is especially recognized for its active protease and peptidase activities toward milk proteins and this strain-specific feature is linked to the release of aromatic compounds, several researches have been carried out in this field (Gatti et al., 2004b, 2008a; De Dea Lindner et al., 2008). A deeper study of the proteolytic enzyme systems and AAs catabolism in *Lb. helveticus* strains was made recently by Broadbent et al. (2011). A comparative genome hybridization analysis was performed to explore the distribution of genes involved in proteolysis and AA catabolism among a bank of *Lb. helveticus* strains isolated from different sources, including PR and GP cheese. Many studies on PR and GP microbiology have been performed by sampling at specific ripening time. More recently the studies on the microbial evolution during the production and ripening of PR and GP cheeses have highlighted the importance of non-starter LAB (NSLAB) in the ripening process (Zago et al., 2007; De Dea Lindner et al., 2008; Gatti et al., 2008b; Neviani et al., 2009; Monfredini et al., 2012; Solieri et al., 2012).

Despite plate culturing techniques are fundamental to isolate microorganisms characteristic of the cheese ecosystem and are indispensable for determining the phenotype of specific strains, their main critical point is the underestimation of the entire microbial community (Head et al., 1998; Marsh, 1999; Giraffa and Neviani, 2001; Santarelli et al., 2008). In fact by these approaches, the most commonly occurring microorganisms are revealed and, among them, only those able to grow to a detectable level by forming colonies in the specific cultural conditions adopted (i.e., viable and cultivable).

Though on one side the development of new culture media can lead to a greater recovery of the cultivable microbiota, on the other side several studies are aimed to recover microorganisms that are either stressed or metabolically active but in a non-cultivable state. Gatti et al. (2006) reported, for the first time, the application of a fluorescence microscopy technique (fluorescence *in situ* hybridization, FISH method) to evaluate microbial viability in natural whey starter for GP. By this method the microbial cell viability correlated with the cell physiological activity, was estimated directly from natural whey starters without any previous isolation step.

In the recent years, the trend is to move toward culture-independent methods that avoid the use of selective cultivation and isolation of bacteria from natural samples, considering the biases related to traditional culture-dependent methods. In fact, the traditional way of studying the microbial composition of a given environment by culture-based methods, is well recognized to fail in characterizing (minor) populations or microorganisms that can be out competed *in vitro* by numerically more abundant microbial species (Hugenholtz et al., 1998) or that can be simply unable to grow *in vitro* (Head et al., 1998). For these reasons, culture-independent approaches have started to be increasingly considered as rapid, sound, and effective alternative methods for the detection of the microbiota also of food products.

Community-level studies are relying more and more on culture-independent methods based on the direct analysis of DNA (or RNA) without any culturing step.

The microbiota of each dairy product has its own history, during which the microbial population structure changes under the influence of continuous shifts in environmental factors occurring during cheese-making and ripening (Coppola et al., 2008). As they are fast, and potentially more exhaustive, culture-independent methods are well suited for analyzing microbial communities over time and may provide the possibility of exploring cheese microbiota in detail, with the major benefit of detecting microorganisms which are difficult to cultivate or uncultivable.

Culture-independent assessment most frequently relies on the analysis of nucleic acids isolated from an entire microbial population, and uses PCR amplification of the target sequences (Quigley et al., 2011). DNA is one of the mainly used target for molecular studies because it can be easily handled and it can fairly resist to degradation. However, DNA-based techniques do not typically permit to distinguish living from non-living microorganisms (Rudi et al., 2005). To exclude detection of non-viable organisms, DNA-based techniques may be combined with an enrichment step (Schaad et al., 1995) or DNA stains, such as ethidium bromide monoazide (EMA) or propidium monoazide (PMA) can be chosen, to differentiate between viable and non-viable organisms (Rudi et al., 2005; Josefsen et al., 2010). In alternative, RNA can be used as a live-cell specific target which allows one to monitor the active microbiota, providing a greater understanding of microbial community structure and functionality (Bodrossy et al., 2006).

In both cases, when using DNA or RNA as target for culture-independent approaches, the outcome is dependent on the extraction efficiency. In fact, DNA may not be recovered from all genotypes, or PCR amplification may be inaccurate. Therefore, in order to characterize whole microbial communities, it is necessary to develop protocols that can be adapted to extract DNA (or RNA) from all different types of microorganisms (Bonaïti et al., 2006). However, some genotypes may remain still undetected due to low species abundance in the substrate, low species availability due to insufficient homogenization of the matrix, inadequate cell lysis that prevents release of nucleic acids, or inhibition of PCR amplification (Jany and Barbier, 2008). Thus, an improving of DNA (or RNA) extraction, which is representative of the total microbial population and is of sufficiently high concentration and purity, can be achieved by complete homogenization of the cheese matrix (Coppola et al., 2006; Parayre et al., 2007), mechanical or enzymatic lysis of cells (Randazzo et al., 2002; Ercolini et al., 2003; Lafarge et al., 2004; Florez and Mayo, 2006; El Baradei et al., 2007; Parayre et al., 2007), protein digestion (Parayre et al., 2007), and DNA precipitation (Duthoit et al., 2003).

Following the extraction of nucleic acids from the cheese matrix, most culture-independent methods rely on PCR amplification of a target sequence. Therefore the selection of a gene or genetic marker that can be used to differentiate a wide variety of organisms, represents a crucial step for molecular assessment of microbial communities (Justé et al., 2008). The most frequently employed targets for identifying bacterial and eukaryote species are respectively the 16S and 26S rRNA-encoding genes (Cocolin et al., 2002; Florez and Mayo, 2006). These are the preferred target region, as they possess both conserved and highly variable domains, which respectively allow discrimination over a wide range of taxonomic levels and serve as annealing sites for universal PCR primers (Justé et al., 2008). Other genes such as the *pheS* (Zago et al., 2009) or the RNA polymerase B subunit (*rpoB*; Martin-Platero et al., 2009) have been targeted to identify bacteria in cheese. In any case, the result of PCR amplification will give amplicons with a sequence which is likely to vary from species to species. The PCR amplicons from different species can be cloned and sequenced to examine the actual diversity of a given community (Delbès et al., 2007; El Baradei et al., 2007), but due to the high costs of this strategy, they are usually discriminated by using gel or capillary separation or by hybridization to specific probes.

The principal techniques that have been used to date to describe microbial communities in cheeses are briefly resumed below.

Polymerase chain reaction-denaturing gradient gel electrophoresis (PCR-DGGE; Myers et al., 1987) and PCR-temporal temperature gradient gel electrophoresis (PCR-TTGE; Yoshino et al., 1991) are based on the separation of PCR amplicons of the same size but with different sequences. In a denaturing acrylamide gel, DNA partially denatures in discrete regions called melting domains, with the melting temperature (*T*_m_) depending on the length of the product, its GC content and the nucleotide sequence. Therefore, amplicons of the same size but with different nucleotide compositions can be separated based on differences in the melting behavior of their melting domains. For PCR-DGGE, the denaturing conditions rely on the use of chemical denaturants (formamide and urea), while for PCR-TTGE, the denaturing gradient is obtained by varying the temperature over time without chemicals, thus generating more reproducible data (Jany and Barbier, 2008; Justé et al., 2008). Further identification of PCR-DG/TTGE is generally obtained comparing profiles to a created database containing migration profiles corresponding to reference strains (Ogier et al., 2002, 2004). However, this kind of database cannot be exhaustive and representative of the actual community analyzed and usually requires constant updates. For this reason, amplicons can be also directly extracted from the DG/TTGE acrylamide gel and sequenced. Unidentified profiles that do not match reference profiles can thus be sequenced and compared to public sequences databases (Jany and Barbier, 2008).

Another technique that relies on electrophoretic separation of PCR products and that has been used for the analysis of microbial communities in cheese, is single-strand conformation polymorphism-PCR (SSCP-PCR; Orita et al., 1989; Lee et al., 1996; Duthoit et al., 2003; Delbès et al., 2007). Like DGGE and TTGE, this method allows separation of different DNA fragments of similar length, but differently from DGGE/TTGE, SSCP separates PCR products based on conformational differences of folded single-stranded products. After denaturation, single-stranded DNA fragments are loaded on a non-denaturing acrylamide gel where a stable secondary structure is formed which is mainly determined by the intermolecular interactions that depend on the nucleotide sequence. Based on the migration of these secondary structures in the gel, products with similar molecular weight can be separated and visualized (Ravnik-Glavac et al., 1994).

Whether the separation of fragments is obtained according to formation of discrete regions of thermal (TTGE) or chemical (DGGE) denaturation or the formation of different single-strand conformation (SSCP), the final output of the analysis will be always a fingerprint. The fingerprint will be made of a number of bands corresponding (in most but not all cases) to as many microbial species and will represent the microbiological identity of the milk, starter, intermediate of production, or cheese analyzed. The final identification of each species can be obtained by the purification and sequencing of each band and by comparison with the available data bases (Coppola et al., 2008).

Besides the above mentioned methods, an increasing number of new methodologies incorporate automatic sequencing systems for laser detection of fluorescently labeled DNA fragments (Santarelli et al., 2008). The most commonly used for cheese microbial community are terminal-RFLP (T-RFLP; Marsh, 1999; Osborn et al., 2000; Rademaker et al., 2005, 2006) and length heterogeneity-PCR (LH-PCR) analysis (Suzuki et al., 1998; Lazzi et al., 2004; Gatti et al., 2008b; Rossetti et al., 2008; Santarelli et al., 2008; Bottari et al., 2010). LH-PCR analysis distinguishes different organisms based on natural variations in the length of 16S ribosomal DNA sequences (Ritchie et al., 2000). Fluorescent end-labeled PCR products are separated by capillary electrophoresis and detected by laser-induced fluorescence with an automated gene sequencer. Following, the relative amounts of amplified sequences originating from different microorganisms can be determined (Suzuki et al., 1998). T-RFLP (Liu et al., 1997) is based on digestion of fluorescent end-labeled PCR products with restriction endonucleases. Amplicon can be labeled, either at one or both 5′ and 3′ ends, by incorporating a dye on either one or both PCR primers. The digested products are separated by electrophoresis using either acrylamide gel- or capillary-based automated sequencer, with laser detection of the labeled fragments. The identification of the different species of a given community is then possible by the use of a database of terminal restriction fragments (TRFs) profiles obtained from reference samples (Dickie et al., 2002). This system only detects the end-labeled TRF of the digested PCR products and their size can be calculated based on the use of DNA size standards that are run simultaneously with the samples. Variation in the presence and location of the restriction sites results in different genotypes having different TRF lengths (Jany and Barbier, 2008).

A technique that differently from SSCP-PCR or T-RFLP permits the collection of elution fractions corresponding to different amplicons, is denaturing high-performance liquid chromatography (DHPLC; Oefner and Underhill, 1995; Xiao and Oefner, 2001). This high-throughput automated detection system allows the direct sequencing of separated amplicons even more easily than with PCR-DG/TTGE methods (Jany and Barbier, 2008). DHPLC was used to study bacterial diversity occurring in natural whey cultures used for cheese manufacture (Ercolini et al., 2008) and to identify yeasts in red smear cheese surfaces (Mounier et al., 2009).

When microbes need to be assessed also in a quantitative manner, quantitative real-rime PCR (qPCR) can be used. Compared to conventional PCR, this method enables an online detection of the PCR product, avoiding the need for post-PCR processing. Usually, DNA amplification is continuously monitored based on the emission of fluorescence. The initial concentration of target DNA is linked to a specific threshold cycle, defined as the cycle number at which fluorescence increases above the background level. Finally, the target DNA is quantified using a calibration curve that relates exact concentrations of template DNA to threshold cycles. Accumulating amplicons can be detected with several chemistries that make use of either fluorescent DNA-intercalating dyes or sequence-specific fluorescent probes (Mackay, 2004; Lievens et al., 2005; Justé et al., 2008). By targeting RNA instead of DNA, reverse transcription-qPCR (RT-qPCR) is also used to study growth dynamics and metabolic activities of microbial species, which is of particular interest for cheese (Ulve et al., 2008; Carraro et al., 2011; Falentin et al., 2012).

Culture-independent methods have proven to be the only ones with the capacity for monitoring the rapid dynamics of microbial communities during cheese manufacture and ripening processes, where microorganisms encounter multiple environmental shifts (Justé et al., 2008). However, culture-dependent and culture-independent methods reveal different images of the same community. Therefore, many authors suggest (Ercolini et al., 2001; Feurer et al., 2004a,b; Florez and Mayo, 2006; Delbès et al., 2007) that using a polyphasic approach, combining both methods, may be worthwhile to obtain a more accurate view of the structure of the microbial community.

Many studies in fact, rely on a combination of both culture-dependent and culture-independent methods and approaches to obtain a more complete picture of the microbiota also of PR and GP. Rossetti et al. (2008), for example, evaluated the species composition and the genotypic strain heterogeneity of dominant LAB isolated from natural whey starter cultures used to GP manufacture, by means of agar plate counting and reverse transcriptase, LH-PCR (RT-LH-PCR). RT-LH-PCR was also formerly used to easily, quickly, and reproducibly assess metabolically active LAB in natural whey starters for GP, in order to verify the effect of several technological parameters on whey starter microbial population (Santarelli et al., 2008). Also Gatti et al. (2008b) used both culture-based and culture-independent techniques to monitor the microbial succession during PR cheese-making. In particular, the intact and lysed cells at different stages of cheese production and ripening were studied by LH-PCR. A polyphasic approach was used as well, to investigate the diversity of dominant LAB population in 12 months ripened PR, combining traditional plating and strains isolation to PCR-DGGE and sequencing of rDNA amplicons (Gala et al., 2008).

A considerable number of molecular-based studies have been dedicated to investigating the microbial composition of a wide variety of cheeses, and to describe as accurately as possible the dynamics, diversity, and behavior of microorganisms during cheese-making and ripening. However, only few studies have been performed using new molecular methods to investigate the microbiota of PR and GP, starting from milk to the ripened cheese.

## MICROBIOTA OF PR AND GP, STARTING FROM MILK TO THE RIPENED CHEESE

Generally speaking the microbiota of the cheese can arise from milk, form starter and also from the environment during ripening. The presence of a hard crust regularly cleaned during ripening makes that this third category is excluded. Thus the microbiota which evolve from curd to ripened GP and PR arises only from raw milk and from natural whey starter. While the first environment (milk) is characterized by a great availability of nutrient and a non-selective temperature (37°C), the latter (naturally acidified whey) is also nutritionally rich but more selective for the pH and temperature condition.

### MICROBIOTA OF RAW MILK

Milk is a nutritionally rich medium in which all microbial groups generally associated with food matrices (pathogenic, spoilage, and useful microorganisms) may be found (Settanni and Moschetti, 2010). Rasolofo et al. (2010) by using two molecular fingerprinting methods, T-RFLP and DGGE, observed that the dominant bacteria in untreated milk belonged to *Staphylococcus*, *Streptococcus*, *Clostridia*, *Aerococcus*, *Facklamia*, *Corynebacterium*, *Acetobacter*, and *Trichococcus*.

Within the useful microorganisms, LAB in milk are usually found at different level and evaluated by bacterial count in agar media. As previous observed (Wouters et al., 2002) plate counts always showed a dominance of LAB cocci over LAB rods, following the general observation that if raw milk is left at room temperature for some time, a microbiota in which mesophilic lactococci predominate will develop.

Lactic acid bacteria reach the milk as contaminants from the udder surface, milking equipment, stable environment and/or transport and filling operations, storage surfaces, and the dairy factory environment (Eneroth et al., 1998). Several LAB species belonging to *Enterococcus*, *Lactobacillus*, *Lactococcus*, *Leuconostoc*, *Pediococcus*, and *Streptococcus* genera are generally recognized in raw milk (Wouters et al., 2002; Franciosi et al., 2009).

Using culture-dependent methods, Coppola et al. (2000) found mesophilic LAB microbiota in PR raw milk at around 10^4^ cfu/ml, whereas De Dea Lindner et al. (2008) and Neviani et al. (2009) found levels approximately 1 log lower. However, this oscillation is compatible with seasonal variation. Isolates by Coppola et al. (2000) were facultatively heterofermentative mesophilic lactobacilli, belonging to *Lb. paracasei* subsp. *paracasei*, and some strains ascribable to *Lb. paracasei* subsp. *tolerans*. A few thermophilic species, such as *Streptococcus *(*St.*)* thermophilus*, *Lb. helveticus*, and *Lb. delbrueckii* subsp. *bulgaricus*, were also isolated. In contrast with Neviani et al. (2009), the authors were not able to recover *Lb. rhamnosus* from milk. Conversely, CAM, used by Neviani et al. (2009), was able to better recover the minority populations, including *Lb. rhamnosus*, than traditional media, such as MRS.

With the same aim, Santarelli et al. (unpublished) used milk plate count agar (MPCA) to analyze raw milk for GP founding mesophilic LAB microbiota ranging between 10^4^ and 10^5^ cfu/ml. Moreover, in order to better recover microorganisms from skimmed milk, Santarelli et al. (unpublished) have adopted an enrichment pre-treatment strategy: aliquots of skimmed vat raw milk were incubated at 30 or 42°C under anaerobic conditions for 12 h. Subsequently, skimmed raw milk treated samples were plated on MPCA and incubated at 30 or 42°C for 72 h under anaerobic conditions. Bacterial isolation from those plates was performed in order to better recover LAB naturally occurring in the milk. Isolates were analyzed by LH-PCR, and in order to confirm results, some strains were also identified by 16S rRNA gene sequencing (Santarelli et al., unpublished).

The majority of these strains provided peaks that according to 16S rRNA gene sequencing corresponded to *St. uberis*, *Lactococcus* (*Lc.*)* lactis* subsp. *lactis* and subsp. *cremoris. *The second most representative isolate was represented by *Lb. delbrueckii* subsp. *lactis* and the third were *Leuconostoc *(*Ln.*)* mesenteroides* subsp. *mesenteroides* and *Lc. lactis* subsp. *cremoris.* Microorganisms belonging to *Enterococcus* genus such as *E. faecalis* and *E. faecium* were also identified in milk. Finally *Lb. helveticus*, *Lb. hilgardii.* *Lb. fermentum*, *Lb. gasseri*, and *Lb. rhamnosus* were differently isolated from milk and milk pre-treated at 30 and 42°C (Santarelli et al., unpublished).

Culture-independent methods were used to study microbiota of milk only recently. The number of viable cells evaluated for the same samples of GP raw milk by Santarelli et al. (unpublished) by fluorescence microscopy was around 100 times higher than values obtained by culture-based method. This data highlight the limit of plate counting technique which was able to reveal, in some samples, no more than 1% of bacteria in milk. Bacterial composition of the same raw milk samples, evaluated by LH-PCR, showed four peaks mainly detected while only two samples showed two peaks. According to the published database (Lazzi et al., 2004; Fornasari et al., 2006; Gatti et al., 2008b), two of those peaks could be attributable to *Lb. delbrueckii* species as well as to *E. faecium* and *E. faecalis*, and to *S. thermophilus* or to *Lc. lactis*, while the others peaks were not identified, since they did not match with any species in the LH-PCR database.

### MICROBIOTA OF NATURAL WHEY STARTER

The starters used to produce GP and PR are undefined cultures which are still prepared in the traditional way by retaining some of the whey drained from the cheese vat at the end of cheese-making. Nowadays, the production of these two PDO cheeses includes cooking at 50–56°C, which occurs before whey drainage. The whey, which is 48–52°C before drainage, is then cooled and maintained at a somewhat controlled temperature until it reaches approximately 30°SH/50 ml and a pH close to 3.5 (Santarelli et al., 2008). Temperature of curd cooking treatment, temperature and modality of whey cooling and the increase of acidity lead to the selection of a characteristic microbiota consisting of thermophilic, aciduric, and moderately heat-resistant LAB (Rossetti et al., 2009; Gatti et al., 2011). Differences in one or more of the many variable parameters can lead to different final cultures. However this modality of preparation warrants the survival of different biotypes useful for the development of the ecosystem itself: a mixture of strains of the same species is necessary for the development of a natural starter with a not well defined composition but with a high technological performance.

Lactic acid bacteria enumeration of natural whey starter was carried out by plate count with different agar media and directly by epifluorescence microscopy both for GP and PR samples, confirming that the bacterial viability in these environments cannot be evaluated only as their capacity to form colonies on MRS, even when acidified. WAM was more useful than MRS but we could suppose that also WAM was certainly insufficient to reproduce the complexity of a natural system where stress factors and equilibrium of population are always in progress.

Generally speaking plate count of natural whey starter, both for GP and PR samples, ranges, in MRS at pH 5.4, from 10^7^ to 10^8^ cfu/ml, and increases in WAM but the total number of cells (mainly viable cells) is even 1 log higher than the cultivable counts (Gatti et al., 2003, 2006, 2008b; Fornasari et al., 2006; Gala et al., 2008; Rossetti et al., 2008; Santarelli et al., 2008; Bottari et al., 2010). Questions remain about the meaning of these dominant population (until 99%) viable but not cultivable. Since these bacteria are not isolable, it is currently not possible to understand if they are potentially important from the technological point of view. Culture-independent methods applied to the study of the microbial ecology of GP and PR natural whey starters revealed that both are dominated by thermophilic LAB belonging to *Lb. helveticus*, *Lb. delbrueckii* subsp. *lactis*, *Lb. fermentum*, and *St. thermophilus* species (Rossetti et al., 2008; Bottari et al., 2010). LH-PCR, and correlated database, were used to recognize and semi quantify the most abundant species, while FISH was a useful indicator of *Lb. helveticus* and *S. thermophilus* viability (Bottari et al., 2006, 2010).

The dominant LAB species found in 24 GP natural whey starter by Rossetti et al. (2008) were *Lb. helveticu*s, *Lb. delbrueckii* subsp. *lactis*, *St. thermophilus*, and *Lb. fermentum*. Combining their presence, the whey cultures could be grouped into four main typologies. The most frequently occurring type contained *Lb. helveticus*, *Lb. delbrueckii* subsp. *lactis*, and *St. thermophilus*. The second most common type contained only the two lactobacilli. Only a minor fraction of the cultures contained *Lb. helveticus* alone or all four LAB species. These data are in agreement with what was recently found by Santarelli et al. (unpublished). DGGE used by Andrighetto et al. (2004) did not allow the amplification of band corresponding to *Lb. delbrueckii*, *Lb. fermentum*, or *S. thermophilus*, which were on the other hand recognized, together with *Lb. helveticus*, by culture-dependent method. This data emphasize the sensitivity of the diverse microbiological methods.

**FIGURE 2:**
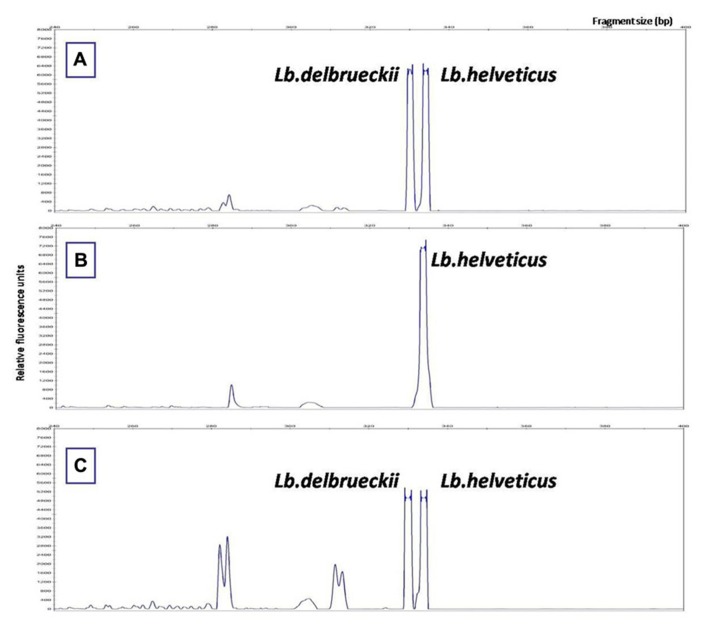
**Microbial profiles of PR natural whey starters revealed by length heterogeneity-PCR: (A)** Presence in comparable percentages of *Lb. helveticus* and *Lb. delbrueckii*, **(B)** presence of *Lb. helveticus* as the dominant species, **(C)** presence of *Lb. helveticus* and *Lb. delbrueckii* percentages comparable to the percentages of other species.

Regarding PR natural whey starter a more complex study was performed: 18 different natural whey starters and the 18 non-acidified wheys from which they arose, were studied using both LH-PCR and FISH (Bottari et al., 2010). LH-PCR electropherograms and FISH revealed *Lb. helveticus*, *Lb. delbrueckii* (reasonably subsp. *lactis*), *St. thermophilus*, and *Lb. fermentum* in almost all samples. The PR natural whey starters could be grouped into three main typologies. The most common typology showed comparable percentages of *Lb. helveticus* and *Lb. delbrueckii*, and the other typologies had *Lb. helveticus* as the dominant species or *Lb. helveticus* and *Lb. delbrueckii* percentages comparable to the percentages of other species (Bottari et al., 2010; **Figure 2**).

### MICROBIAL EVOLUTION FROM CURD TO RIPENED CHEESE

When the natural whey starter is added to the partially skimmed milk in the vat, their microbiota is mixed. The amount of natural whey starter added is approximately 3%, thus microbial equilibrium between natural whey starter and milk can be about 100:1 in favor of the first but it can also drastically change in one or other direction.

Considering the different origin and the different meaning during cheese production and ripening, LAB of GP and PR can be also defined as starter LAB (SLAB) and NSLAB. The first are involved in acid production during manufacture and contribute to the ripening process while the latter do not contribute to acid production during manufacture, but generally play a significant role during ripening (Beresford et al., 2001). It is very common to distinguish them according to their origin, the SLAB mainly arise from natural whey starter and NSLAB mainly form milk. However the link between the milk and the whey, with which natural whey starter is prepared, is very close and for this reason it is possible to find NSLAB (species not involved in acidification) also in natural whey starter and SLAB (species involved in acidification) also in milk.

During GP and PR production, the fermentation process is initiated by inoculating milk with natural whey starter mainly composed by SLAB that, even if may partially grow in vat, mainly develop during acidification, after curd extraction. Here they grow and survive under acidic, oxidative, osmotic and nutritional stress which increase during salting (around 20 days) and ripening (at least 9 months for GP and 12 months for PR). The variations of temperature, salt concentration, water content, and pH are modulated by each cheese-making process. The increasingly adverse conditions reduce SLABs viability until a sizable fraction of the starter cells undergo autolysis. For this reason, SLABs are involved not only in lactose depletion and curd acidification, but also in the production of a considerable amount of the intracellular aminopeptidases, which are released as a consequence of bacterial lysis and are active throughout ripening (De Dea Lindner et al., 2008). The release of intracellular enzymes and other cellular components into the cheese matrix are determined in the final proteolysis of cheese casein, producing AAs that may serve also as substrate for the growth of NSLAB microbiota.

On the contrary, NSLAB are not part of the normal starter, coming from untreated milk, they are present at low concentrations in curd where metabolic antagonism of SLAB prevent or severely reduce their multiplication. Their high toleration to the hostile environment, salt concentration, low moisture, 4.9–5.3 pH values, low temperatures, a deficiency of nutrients, allow to NSLAB to develop, mainly after brining (Fox and McSweeney, 2004; Cogan et al., 2007; Settanni et al., 2012). Increasing of about four to five orders of magnitude within a few months, they can have a key role in determining curd maturation and final characteristics of cheese (Settanni et al., 2012).

Only the use of new molecular techniques has allowed to prove these observations which were, until few years ago, for GP and PR, always hypothesized and only partially confirmed. The main obstacle was due to the possibility to use only culture-depended methods and recognize LAB species only after difficult and time expensive identification of many isolates. With these difficulty, Coppola et al. (1997, 2000), however, were able to recognize the main species characteristic of PR without being able to highlight their evolution.

Culture-independent methods have made possible the study of the microbiota of PR and GP. In this context, a more recent work has led to new findings that advance the understanding of microbial dynamics in PR (Gatti et al., 2008b). A distinguishing trait of this research was the availability of 16 twin cheese wheels, providing representative samples of the subsequent stages of the same cheese-making process. LH-PCR was used and a particular sample preparation method allowed the evaluation of both whole and lysed cell dynamics during cheese-making and ripening. The novelty of this research was also the evaluation of the most important macroscopic microbiological aspects in a ripened cheese where viable, not-viable, and lysed microbial cells coexist. The results of this particular sampling confirmed that the thermophilic microbiota of natural whey starter for PR is mainly composed of *Lb. helveticus* and *Lb. delbrueckii* subsp. *lactis*. The identification of other species grown in the curd has not been possible maybe due to the limit of detection. Whole *Lb. helveticus* and *Lb. delbrueckii* subsp. *lactis* cells were found to be dominant until the second month of ripening, although an increasing number of them underwent autolysis. One month after brining, *Lb. rhamnosus* or *Lb. casei* and *P. acidilactici* were detected. In this stage of PR production, lysed cells of *Lb. helveticus* and *Lb. delbrueckii* species were revealed together with intact ones, and it is possible to hypothesize that the whole cells were under quiescent conditions and may be viable, but non-cultivable and still not lysed. Non-attributable peaks were found in the LH-PCR profiles of both lysed and whole cells, suggesting the presence of other unknown, non-cultivable species. After 6 months of ripening, the same species were found, although none of them seemed to be dominant. Interestingly, in this stage of ripening, NSLABs (*Lb. rhamnosus* or *Lb. casei* or *Lb. plantarum*), which also seemed to increase, undergo an autolysis process. Between the 6th to the 20th month of ripening, no microbial evolutionary changes were observed.

Another study offered a global view of the possible contribution of total, viable, cultivable, and lysed bacterial cells during the ripening of PR (De Dea Lindner et al., 2008). In this work cells lysis was evaluated indirectly measuring the activity of the enzymes released considering in particular aminopeptidase activities involved in cheese ripening. It was observed that, during ripening, a strong decrease in the total bacterial population corresponded to an increase in the activity of four of the six peptidases evaluated.

The presence of SLABs and their enzymes is not sufficient to explain flavor formation in raw-milk cheeses. In fact, raw-milk cheeses are considered to have better flavor than those made from pasteurized milk, indicating that the raw milk microbiota (including NSLAB) and perhaps heat-sensitive enzymes have an effect on flavor.

To study NSLAB evolution during PR ripening, 66 *Lb. rhamnosus* strains isolated during the same cheese-making session were genotypically characterized by Bove et al. (2011). Interestingly, the intraspecies heterogeneity they found seemed to be correlated to the ability of bacteria to adapt to specific environmental and technological conditions. The highest number of different biotypes was isolated in the first period of cheese maturation; in particular, they were found in the curd after 6 and 48 h and in the cheese after brining. After this time, these biotypes disappeared, perhaps because of increasingly unfavorable conditions. In contrast, other biotypes were isolated only at the end of ripening. Unfortunately, this finding might be due to the lower number of isolates obtained from the first samples or to the difficulty of isolating these biotypes. The majority of the strains, however, belonged to biotypes that are present in cheese from the first or second month of ripening until beyond 10 months. One of these is most frequently isolated and therefore might be considered the dominant strain throughout the whole ripening process (Bove et al., 2011).

Also Solieri et al. (2012) verified an interesting biodiversity among the same species. In particular a culture-dependent multiphasic approach was used to characterize NSLAB isolated from different PR cheeses. Different months of ripening (from 7 to 23) and different dairies were considered. *Lb. rhamnosus* and *Lb. paracasei* were the species most frequently associated to the isolates and among them an intraspecific diversity was revealed. Also in this case, both for *Lb. rhamnosus* and *Lb. paracasei*, ripening time appears to play a role in composition of cultivable NSLAB in cheese.

The detection of biotypes that correlate with specific moments in cheese ripening or differential development throughout this process suggests that these strains may have specific roles closely linked to their peculiar technological properties; however, this aspect should be more deeply examined in future studies.

Regarding GP population dynamics, lactobacilli were selectively enumerated by colony hybridization, during an experimental cheese-making and ripening (Zago et al., 2007). In particular, a specific detection of *Lb. helveticus*, *Lb. delbrueckii* subsp. *bulgaricus* and *Lb. delbrueckii* subsp. *lactis* was performed counting bacterial colonies grown on Hybond N+ membranes layered on MRS solid medium with DNA probes that were specific for the identification of *Lb. helveticus* (de los Reyes-Gavilàn et al., 1992), *Lb. delbrueckii* (Delley et al., 1990), and *Lb. delbrueckii* subsp. *lactis* (Giraffa and Mora, 1999).

This cultured-dependent approach, confirmed by the identification of many isolates, indicated that *Lb. helveticus* was the dominant species in the natural whey starter and during the early stages of cheese-making, whereas *Lb. delbrueckii* subsp. *lactis* predominated later, after 2 months of ripening. Sequential hybridizations with different DNA probes specific for *Lb. delbrueckii* and *Lb. delbrueckii* subsp. *lactis* indicated that approximatively the 99% of the *Lb. delbrueckii* population belonged to *Lb. delbrueckii* subsp. *lactis*. *Lb. delbrueckii* subsp *bulgaricus*, although is a species typical of many dairy products such as fermented milks and other cheeses (Rault et al., 2009; Hao et al., 2011), does not adapt easily to GP and PR environment. At advanced stages of this experimental GP cheese ripening, mesophilic lactobacilli were recovered in high numbers, and further identification determined that they belonged mostly to *Lb. casei* or *Lb. paracasei* and *Lb. rhamnosus* (Zago et al., 2007). PCR fingerprinting of these mesophilic lactobacilli revealed a high strain heterogeneity linked to their different origins: raw milk, the dairy environment, or the cheese-production environment (Zago et al., 2007).

Differently from Zago et al. (2007), who performed the research on experimental cheese, not-yet-published researches were carried out considering a complex and varied sampling of PDO GP cheeses (Santarelli et al., unpublished; Pogacic et al., unpublished). The first research evaluated six GP cheese-making processes in six different dairies from the PDO production area. In this way it was possible to evaluate not only the trend of microbial dynamics but also differences among productions (Santarelli et al., unpublished). By means of LH-PCR, profiles of the bacterial community were obtained and compared. Identification of SLAB such as *Lb. helveticus* and *Lb. delbrueckii* subsp. *lactis* in the first hours of production confirmed their origin from natural whey cultures and the well-known role in curd acidification. However their presence, as a non-cultivable state, up to 13 months of ripening suggested a different unknown role in cheese ripening. As observed for PR, SLAB were the main species during the acidification steps of GP production and NSLAB were able to grow after brining, becoming dominant during ripening. Interestingly in this research it was demonstrated that the LAB able to grow under the specific cheese environmental conditions during ripening, could arise both from raw milk and natural whey starters. For the first time ecological indices were used to study microbial ecology during manufacture and ripening. The vat skimmed raw milk ecosystem showed higher diversity, evenness and richness of bacterial community compared to the natural whey starter ecosystem. Among cheese ecosystems, diversity, evenness, and richness showed changing trends. Differences in the qualitative and quantitative rate of cell lysis of SLAB and NSLAB population were found among 2-month cheeses with potential effects on ripening process and flavor development (Santarelli et al., unpublished). Moreover, in the same research, the rates of LAB lysis were monitored by determining the free DNA released from dead or damage cells in 2-month cheeses. Lysis rates were calculated as the sum of the areas of all peaks in the LH-PCR profiles of free DNA fraction of each sample. The relative percentage of cell lysis was calculated by measuring the individual and total peak area. Free DNA from lysed *Lb. helveticus* and *Lb. delbrueckii* subsp. *lactis* was found in all the 2-month cheeses, confirming that after acidification, starter lysis occurs. Contrarily to Gatti et al. (2008b) that did not reveal the presence of whole and lysed cells of *Lb. fermentum* during PR cheese ripening, *Lb. fermentum* lysed cells were observed and for many cheese samples high relative percentages were reported. The extent of cell lysis in cheese affects the proteolysis rate and the length of ripening (Cogan and Beresford, 2002). Autolysis of different species was shown to induce the release of different enzymes that determine the degradation of casein derivatives during ripening (Kunji et al., 1996). The effect of this process may highly affect the texture and flavor of cheese in each dairy. A relationship between the rate and the type of LAB cells autolysis and flavor development in ripened GP is the topic of a research which is going to be concluded (Lazzi et al., unpublished).

Finally, culture-dependent and culture-independent methods were applied to deeper investigate the microbial ecology and the dynamic of autochthonous strains of LAB during ripening phases of GP cheese to evaluate the differences between different localities of cheese manufacturing for the strain biodiversity of autochthonous LAB (Pogacic et al., unpublished). In this research, LH-PCR was used as consolidated reference culture-independent method in order to highlight the importance of using both the approaches to know the microbiota of raw-milk, long-ripened cheeses.

## CONCLUSION

Natural ecosystems such as fermented foods should be viewed as much as possible as wholes. In the case of GP and PR, the main sources of the most valued microbial biodiversity that produces the unique taste and aroma of these cheeses are the raw milk, the typical way of milk skimming and the use of natural whey starter are. The dynamics of growth, survival, and biochemical activity of these microorganisms in the cheese are the result of stress reactions in response to physical and chemical changes in the food matrix and of interactions among the single bacterial colonies that are part of cheese microbiota. To understand the real role of these interactions and the impact of cells presence and activity, different analytical strategies are probably necessary. Even if an holistic approach is difficult to apply, at least a multiphasic approach to study food fermentations would improve our understanding of these processes. This approach is, in our opinion, essential for studying raw-milk, long-ripened cheeses where contribution of viable, viable but not cultivable, damaged, and lysed SLAB and NSLAB cells is different depending on the different steps of cheese-making and ripening. The methods described in this article cannot be considered exhaustive, they are continuously improved and they can be replaced by others more and more performing. However, they allow a depiction of the studied cheese-making processes, representing important tools to study the microbial ecology of GP and PR during transformation from milk to ripened cheese. Plating analysis should not be considered as overcome and neither to be used separately; in fact, the combination of the results obtained by culture-dependent and culture-independent methods allows us to carefully profile microbial dynamics. They permit the identification of specific populations which can be also selected for their particular characteristics and be used as starter cultures to improve the sensory profile of other long-ripened cheeses.

## Conflict of Interest Statement

The authors declare that the research was conducted in the absence of any commercial or financial relationships that could be construed as a potential conflict of interest.

## Footnotes

1 www.parmigiano-reggiano.it

2 www.granapadano.com
